# *Bacillus subtilis* Swarmer Cells Lead the Swarm, Multiply, and Generate a Trail of Quiescent Descendants

**DOI:** 10.1128/mBio.02102-16

**Published:** 2017-02-07

**Authors:** Lina Hamouche, Soumaya Laalami, Adrian Daerr, Solène Song, I. Barry Holland, Simone J. Séror, Kassem Hamze, Harald Putzer

**Affiliations:** a Institut de Biologie Physico-Chimique, UMR 8261, CNRS-Université Paris Diderot, Paris, France; b Matière et Systèmes Complexes, UMR 7057, CNRS-Université Paris Diderot, Paris, France; c Institute for Integrative Biology of the Cell, UMR 8621, CNRS-University Paris-Sud, Orsay, France; d Lebanese University, Faculty of Science, Section 1, Beirut, Lebanon; Tel-Aviv University; University of Washington

## Abstract

Bacteria adopt social behavior to expand into new territory, led by specialized swarmers, before forming a biofilm. Such mass migration of *Bacillus subtilis* on a synthetic medium produces hyperbranching dendrites that transiently (equivalent to 4 to 5 generations of growth) maintain a cellular monolayer over long distances, greatly facilitating single-cell gene expression analysis. Paradoxically, while cells in the dendrites (nonswarmers) might be expected to grow exponentially, the rate of swarm expansion is constant, suggesting that some cells are not multiplying. Little attention has been paid to which cells in a swarm are actually multiplying and contributing to the overall biomass. Here, we show *in situ* that DNA replication, protein translation and peptidoglycan synthesis are primarily restricted to the swarmer cells at dendrite tips. Thus, these specialized cells not only lead the population forward but are apparently the source of all cells in the stems of early dendrites. We developed a simple mathematical model that supports this conclusion.

## IMPORTANCE

Swarming motility enables rapid coordinated surface translocation of a microbial community, preceding the formation of a biofilm. This movement occurs in thin films and involves specialized swarmer cells localized to a narrow zone at the extreme swarm edge. In the *B. subtilis* system, using a synthetic medium, the swarm front remains as a cellular monolayer for up to 1.5 cm. Swarmers display high-velocity whirls and vortexing and are often assumed to drive community expansion at the expense of cell growth. Surprisingly, little attention has been paid to which cells in a swarm are actually growing and contributing to the overall biomass. Here, we show that swarmers not only lead the population forward but continue to multiply as a source of all cells in the community. We present a model that explains how exponential growth of only a few cells is compatible with the linear expansion rate of the swarm.

## INTRODUCTION

Bacteria are unicellular organisms, but they can accomplish certain tasks only when individuals act together. For surface-dwelling organisms, it is a major challenge to migrate from one location to another. Most bacteria can develop swarming motility that results in a rapid (2 to 10 mm/h) coordinated translocation of a microbial community across a surface (1–3). In nature, this multicellular behavior can be viewed as a territorial expansion, often preceding the formation of a sessile biofilm (4). Interestingly, both swarming and biofilm-associated bacteria can develop adaptive tolerance to antibiotics (5). Under laboratory conditions, depending upon the species and the composition of the medium, swarming migration takes on a wide variety of forms, from largely featureless to highly branched dendritic patterns (as studied here). Nevertheless, all forms of swarming share several characteristics, including cooperative movement in thin films, hyperflagellation, secretion of a wetting agent, and a high-density population of specialized swarmers. These are localized to a narrow zone at the swarm front that in small groups display high-velocity whirls and vortexing. The swarmers usually form a monolayer a few millimeters wide that, in most cases, rapidly switches to multilayered growth behind the swarmer zone that finally constitutes the bulk of the swarm biomass (for reviews, see references 6 and 7). Bacterial swarmers have altered transcription profiles; however, these have provided only limited insights into the specific mechanisms associated with the swarming process (8–12).

In *Proteus mirabilis*, cyclical swarms involve periods of consolidation, when migration halts. At this point, swarmers dedifferentiate to single-cell swimmers that then undergo extensive growth, combined with suppression of division to produce a new generation of the characteristic filamentous, hyperflagellated swarmers. These then migrate to the colony front to initiate a new round of swarming, with the implicit assumption that further growth is arrested during the swarming process (11, 13). Thus, the majority of cells in these communities are likely produced during the consolidation periods. Such cyclical waves of swarming have not been observed in most other species, and surprisingly, little or no attention has been paid to the origin of the bulk of new biomass in other swarm communities.

*Paenibacillus vortex*, a soil bacterium extensively studied for its swarming behavior (14–16), displays coexisting subpopulations referred to as hyperflagellated “explorers” and “builders,” constituting the majority of the swarming population (17). This is reminiscent of the swarmer and nonswarmer populations, based on single-cell *in situ* expression of specific genes that we previously identified in *Bacillus subtilis* (18). Roth et al. (17) analyzed liquid cultures inoculated with explorer or builder cells and concluded that the explorers are metabolically less active (reduced levels of ATP) and apparently have a reduced growth rate compared to that of the builders. In line with the *P. mirabilis* studies, these observations supported the general view that the minority swarmer cell population leads and drives the swarming community but that the cells following behind produce the biomass, due to their higher growth rate.

However, to our knowledge, no direct examination and comparison of the growth status of swarmer and nonswarmer cells *in situ* has been reported for any organism.

In *B. subtilis*, swarming migration of the largely multilayered mass of cells from a central inoculum on a rich medium, LB (19), is maintained at a constant rate throughout. Similarly, on synthetic B medium, characterized in this case by its highly branched, monolayered dendritic pattern (18, 20, 21), swarm expansion also proceeds at a constant rate. Moreover, the population density of the initial dendrites, including the 1- to 2-mm tip region containing the hypermotile swarmers, also remains relatively constant. This behavior is unusual for a bacterial population, since it does not appear compatible with exponential growth of the whole population, although nutrients under these conditions are not apparently limiting (see below). It is thus important to understand whether and how the growth characteristics of subpopulations within the community are significantly different. However, as in other bacteria, attempts to measure the precise growth status of cells in the swarming community have not been reported.

In our model system, based on a carefully optimized protocol, *Bacillus subtilis* swarms rapidly (up to 10 mm h^−1^) over a soft-agar synthetic medium, forming a hyperbranched, dendritic pattern that covers a petri dish in a few hours (20). This experimental system was also successfully used recently for the analysis of kin discrimination among various *B. subtilis* isolates (22). The employment of such a defined medium combined with a small inoculum of cells may better mimic slow-growth conditions in nature and enables a highly reproducible spatiotemporal development of the swarm. This proceeds as a series of distinct morphological and genetically defined stages (20, 21, 23) according to a highly predictable timing schedule. A few minutes prior to the initiation of migration, a spreading zone of surfactin (24) emanates from the multilayered mother colony (MC; the site of inoculation). This is rapidly followed by an outward burst of several monolayered, small, budlike structures from the edge of the MC, which then extend to form the primary dendrites. These continue to elongate as a monolayer up to at least 1.5 cm. Finally, a switch to multilayering occurs from the base of the dendrites, spreading outwards with the formation of fractallike biofilms (20, 21, 23). This system enables spatiotemporal *in situ* gene expression analysis in single cells, including the homogenous population of hyperflagellated swarmers. These are precisely demarcated within the final 1 to 2 mm at the tip of each dendrite (18), with other distinguishing characteristics that include collective swimming as high-velocity swirling vortices and a reduced cell size (18). In contrast, on a rich medium like LB, *B. subtilis* swarming involves extremely rapid expansion of an amorphous, multilayered mass of cells (19). Nevertheless, the hypermotile swarmers also appear to be restricted to a 1-mm monolayer at the leading edge, while initial short-lived dendrites at the front apparently fuse laterally, thus suppressing dendrite formation (25). Concomitantly, the very early switch to multilayered growth (biofilm formation) on LB considerably shortens the exploitable time for single-cell *in situ* analysis (in monolayers), which represents the actual swarming phase.

We have previously proposed that the nonmobile cells in a *B. subtilis* swarming community extend dendrites by multiplication (18), in line with the perception that swarmers might drive the community at the expense of growth (26). In order to test this hypothesis and to determine which cells within the migrating community are multiplying, we studied the expression of key genes involved in DNA replication and protein synthesis at the single-cell level using green fluorescent protein (GFP) fusions. We further tracked cellular growth using fluorescent d-amino acids (FDAAs), which allow the labeling of sites of active peptidoglycan (PG) synthesis (27). Surprisingly, we found that while the swarmers were apparently multiplying, the pattern of biosynthetic activity in the rest of the dendrite showed a progressive shutting down, until at the base, DNA replication, protein translation, and cell wall synthesis were minimal. We present a mathematical model that describes the expansion of the community where exponential growth is restricted to the swarmer population. Supporting the experimental findings, the model predicts that the great majority of the cells in the dendrites are descendants of swarmers.

## RESULTS

### The experimental system.

**FIG 1:**
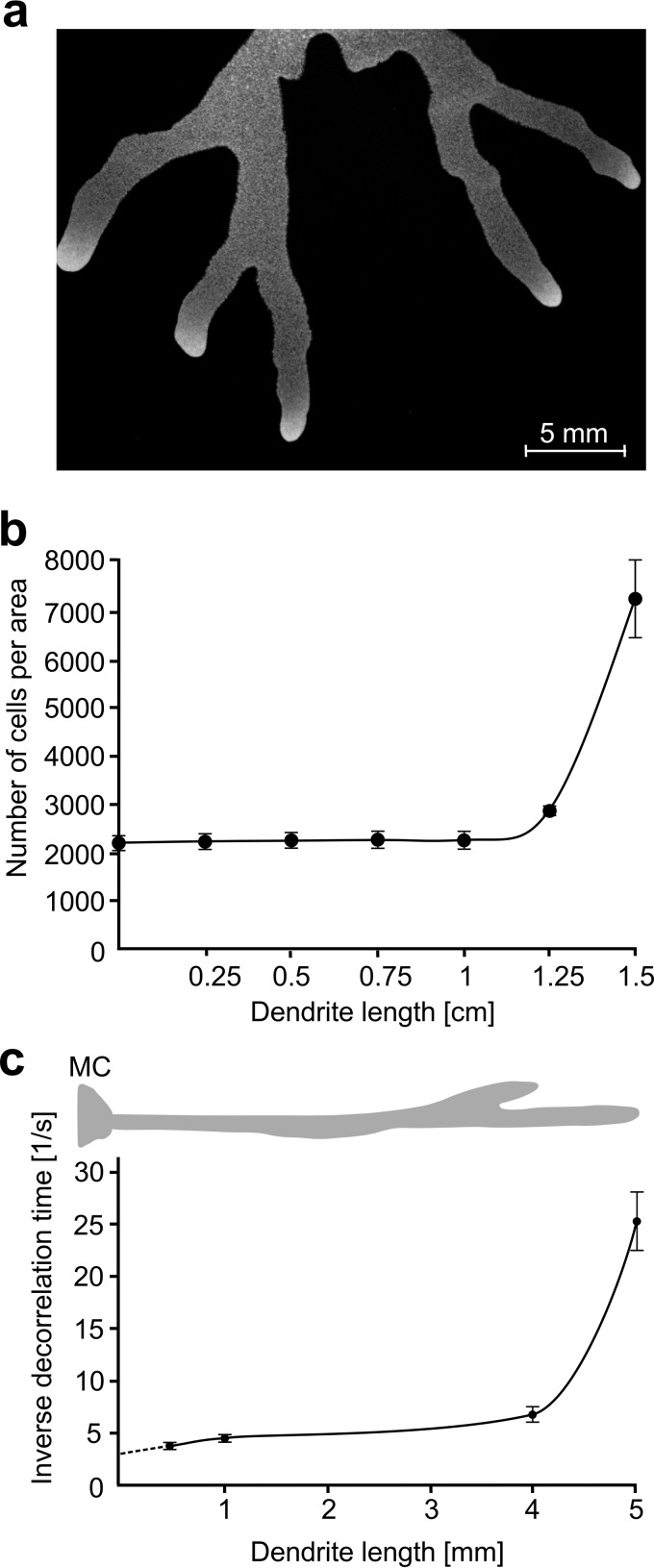
Physical swarm characteristics. (a) Stereomicroscope photograph of a typical monolayered *Bacillus subtilis* dendritic swarm (strain OMG981) expressing GFP (ribosomal protein *rpmGB-gfp* fusion described in the section “Translational activity across the swarming community”). (b) Number of cells per area (0.0516 mm^2^) at indicated distances from the edge of the mother colony to the tip of the dendrite. (c) Inverse decorrelation time plotted against the position of the cells along a 0.5-mm long dendrite. The decorrelation time (calculated from a 45-image/s film) gives a quantitative measure of the mobility of the bacteria in a short period of time. MC, mother colony. Error bars represent the standard deviations of the means.

For this study, we used a derivative of the laboratory strain 168, SSB2026, which carries wild-type alleles for the *sfp* (required for activating surfactin) and *swrA* genes, both important for swarming (28). SwrA promotes robust swarming but is only required when the humidity falls below 50% (18). We have previously shown that strain SSB2026 exhibits swarming characteristics very similar to those of the nondomesticated strain 3610 (18). We preferred this derivative of the laboratory strain over the nondomesticated wild type since, in the latter, the dendrites branch earlier and the distance over which the cells remain as a monolayer is shorter. The major characteristics of the swarm formed by SSB2026 were determined while the bacterial community advanced as a monolayer of cells (up to 1.5 cm) (Fig. 1a). At 30°C, the speed of translocation was measured at different stages of dendrite development and remained stable at 1.6 to 2 mm/h. As observed previously (18), the population density was relatively constant throughout the stem of the dendrites, which we shall refer to as composed of “the stem cells.” The population density increased sharply (3.3-fold) at the tip of the dendrite, comprising the hyperflagellated swarmer cell population (Fig. 1b). The high population density is associated with hypermotility of the cells, which move as rapidly swirling groups (see Movie S1 in the supplemental material). The swarmer cells always occupy approximately 1 mm at the very tip of each dendrite. This remains essentially invariable from the formation of the buds to the arrival of dendrites at the edge of the plate. A decorrelation time analysis (see Materials and Methods) also shows that the fastest-moving bacteria are restricted to the foremost millimeter of the advancing community (Fig. 1c). On average, the width of the extending dendrites is also about 1 mm.

10.1128/mBio.02102-16.1 MOVIE S1  The swarmer population at the edge of an advancing dendrite (*B. subtilis* strain SSB2026, used in this study) was filmed *in situ* at 40 images/s with a 63× air objective on a Zeiss AxioImager M1 microscope. Download MOVIE S1, MOV file, 2.9 MB. Copyright © 2017 Hamouche et al. 2017 Hamouche et al. This content is distributed under the terms of the Creative Commons Attribution 4.0 International license.

The generation time in a liquid culture in B medium for SSB2026 is 110 min at 30°C, but it is not technically feasible to measure the cell division rates of the rapidly moving swarmer cells directly under the microscope. Since the swarm advances at a constant rate, the cells cannot all be growing exponentially, and we assumed that some cells are growing and others are not. To understand how multiplication may depend on the localization of a single cell in the community, we first studied the expression of key genes involved in DNA replication and protein synthesis.

### Replication profile along the dendrite. (i) DnaA.

The replication initiator protein DnaA is highly conserved in bacteria (29) and initiates replication by unwinding the DNA (30). In *B. subtilis* growing exponentially in liquid culture, DnaA foci colocalize with the chromosomal origin of replication (*oriC*) (31–33), but this colocalization decreases and/or disappears in nonreplicating cells (31).

**FIG 2:**
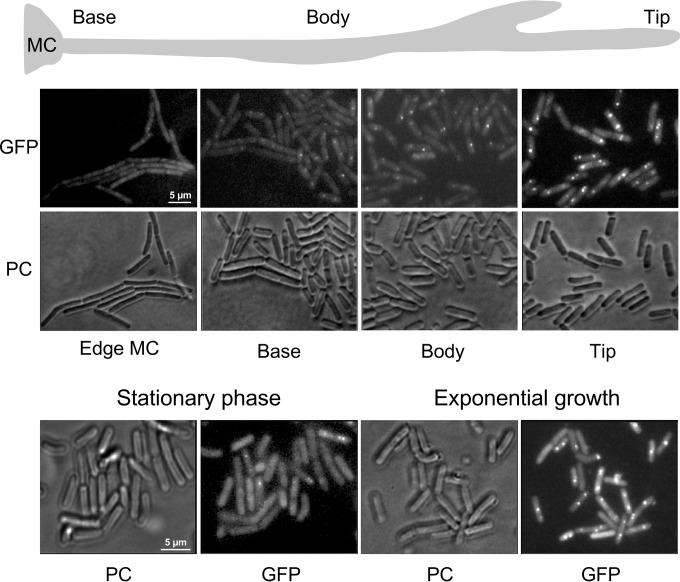
Expression and localization of the replication initiator protein DnaA. (Top) Cells from a 1.5-cm swarm producing a fluorescent GFP-DnaA fusion protein (strain SSB2041) were removed from the indicated positions and analyzed by fluorescence microscopy (×100). Panels labeled “PC” show the same cells imaged by phase-contrast microscopy. (Bottom) GFP-DnaA expression in the same strain grown in liquid culture is visualized. Cells were taken either during exponential growth or from the stationary phase and analyzed by fluorescence microscopy under the same conditions as the swarming cells. MC, mother colony.

In order to analyze DnaA localization in individual cells of the swarming community, we expressed an N-terminal GFP-DnaA fusion protein under the control of a xylose-inducible promoter located in a plasmid integrated in single copy in the chromosome. Cells from at least 40 dendrites were removed at different positions along the ~1.5-cm-long monolayers, immobilized, and analyzed by fluorescence microscopy. As shown by the results in Fig. 2, single DnaA fluorescent foci were generally found near the center of cells, with some cells showing two foci. The intensity and frequency of the foci in tips were clearly higher than in cells taken from the mother colony, the base, and the body of the dendrite. The visual result was fully corroborated by quantitative single-cell measurements (see Fig. S1 in the supplemental material). These results are consistent with the notion that the swarmers at the front of the migrating community are the cells initiating replication the most frequently.

10.1128/mBio.02102-16.2 FIG S1  Expression and distribution of the replication initiator protein DnaA. (a) Mean fluorescence intensity of DnaA foci observed in individual cells localized at the base, body, and tip of the swarming community. (b) Percentages of cells that show a diffuse DnaA expression pattern and no visible GFP-DnaA foci. Error bars represent the standard deviation of the mean. AU, arbitrary units. Download FIG S1, PDF file, 0.1 MB. Copyright © 2017 Hamouche et al. 2017 Hamouche et al. This content is distributed under the terms of the Creative Commons Attribution 4.0 International license.

In liquid cultures, we found strongly fluorescent DnaA foci similar to those seen in the dendrite tip only in exponentially growing cells (92%, 148 of 161 cells analyzed), while in a stationary-phase liquid culture, the majority of cells showed a diffuse DnaA distribution and only a few cells (18%, 32 of 179 cells analyzed) showed visible but faint foci (Fig. 2, bottom). We therefore examined our images from the swarming community with respect to the percentage of cells where GFP-DnaA was dispersed and clear foci were absent. Close to the edge of the mother colony, where most of the cells are in a long-chain form, 80% of cells belonged to this category. This percentage was 70% at the base of the monolayer and decreased progressively toward the tip of a dendrite, where only 17% of cells showed a diffuse DnaA distribution, while 75% had easily detectable foci (Fig. 2; see also Fig. S1b in the supplemental material).

The DnaA localization data thus clearly suggested that the swarmer cells are the most actively replicating cells within the community and that DNA replication in most of the stem cells of the dendrite is largely arrested or at least severely reduced.

### (ii) DnaN.

We chose DnaN, the β-2-sliding clamp of DNA polymerase (34), as a second independent reporter for active replication. GFP-DnaN forms foci that accompany replication fork progression in actively dividing cells (35, 36), while arrest of replication initiation leads to the disappearance of GFP-DnaN foci and dispersal of fluorescence throughout the cell (36). We looked for this phenotype to detect actively replicating cells within the swarming community. To achieve this, the *dnaN* gene was replaced by a *gfp-dnaN* fusion construct that expresses a previously described fully functional GFP-DnaN protein (37) from its endogenous locus.

**FIG 3:**
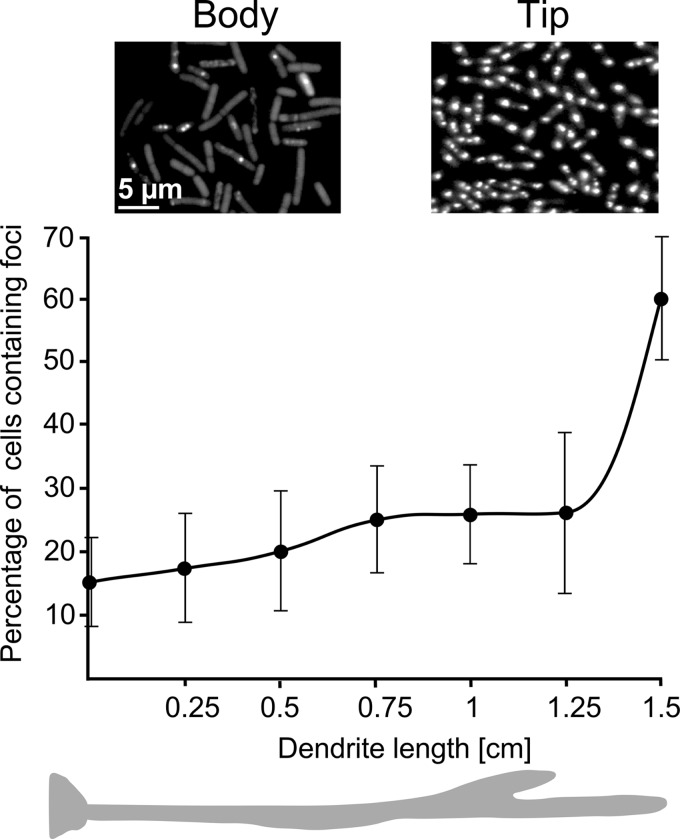
Spatiotemporal expression of the DnaN subunit of DNA polymerase. The images taken *in situ* show the production of a GFP-DnaN protein fusion in cells located in the dendrite body and the tip of the swarming community (strain SSB2022). The graph shows the percentages of fluorescent foci in individual cells measured every 2.5 mm along the dendrite. Error bars represent the standard deviations of the means.

In liquid culture, we observed that more than 90% of exponential-phase cells had bright GFP-DnaN foci. However, when grown for 4 h into stationary phase, GFP-DnaN was completely dispersed in most of the cells, and only a few retained weak foci (see Fig. S2 in the supplemental material). In order to study the production of GFP-DnaN in the swarming community, high-magnification fluorescent images were taken *in situ* along 1.5-cm- and 2-cm-long dendrites in this experiment. Different areas of the monolayer containing 400 to 800 cells were then analyzed (see Materials and Methods). Strongly fluorescent foci, mostly in the center of the cell, were only observed in the swarmer cell population at the tip of a dendrite (Fig. 3; see also Fig. S2). In contrast, the stem cells, starting with those immediately behind the swarmer population (1 to 2 mm from the migration front), remarkably had only weak foci. Moreover, GFP-DnaN was almost totally dispersed in the cells at the base of dendrites and at the edge of the mother colony (see Fig. S2). Surprisingly, this indicates that DNA replication rapidly ceases in cells immediately outside the advancing zone of the highly mobile swarmer cells. To confirm this, we quantified the percentages of cells containing visible foci by taking images *in situ* every 2.5 mm along 1.5- to 2-cm dendrites. The number of focus-containing cells increased gradually from 16% to about 25% from the base toward the distal end of the dendrite but then increased sharply to 60% with, also, markedly increased intensity within the last 2 mm at the tip (Fig. 3). We conclude that only the swarmer cells, i.e., within the first 1 to 2 mm at the front of the migrating community, are replicating DNA actively. Replication of the largely immobile stem cells making up most of the biomass of the dendrite is arrested or at least strongly reduced.

10.1128/mBio.02102-16.3 FIG S2  GFP-DnaN expression in the swarm and liquid culture. (Top) *In situ* fluorescence microscopy images (×100) of a 1.5-cm swarm expressing a fluorescent GFP-DnaN fusion protein (strain SSB2022). The images taken in the body of a dendrite and at the tip of the dendrite are identical to those shown in Fig. 3. (Bottom) Control experiment where strain SSB2022 was grown in liquid culture (B medium). Cells were taken either during exponential growth or from stationary phase, and GFP-DnaN expression analyzed by fluorescence microscopy under the same conditions as the swarming cells. Images labeled “PC” show the same cells imaged by phase-contrast microscopy. Download FIG S2, PDF file, 0.4 MB. Copyright © 2017 Hamouche et al. 2017 Hamouche et al. This content is distributed under the terms of the Creative Commons Attribution 4.0 International license.

### Translational activity across the swarming community.

**FIG 4:**
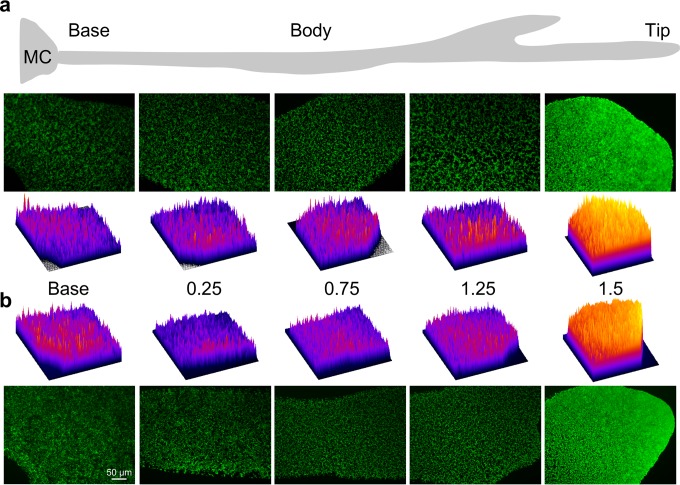
Semiquantitative *in situ* analysis of rRNA and protein production along the dendrites. (a) GFP levels expressed from the dual *rrnB* promoters (strain SSB2020) were visualized by fluorescence microscopy at low resolution (×15) throughout an entire 1.5-cm monolayered dendrite. Selected images covering different regions of the community are shown. Semiquantitative analysis of fluorescence intensity per cell was carried out every 2.5 mm as indicated. Purple represents minimal expression, while the red-to-yellow gradient reflects the highest levels of fluorescence intensity. (b) GFP levels controlled by the transcriptional signals of the *rpmGB* gene encoding ribosomal protein L33 (strain OMG981). Analysis was as described for panel a.

Actively dividing bacteria in liquid cultures devote more than half their energy to protein synthesis. The number of ribosomes manufactured increases with the square of the growth rate (38, 39). Ribosomal RNA synthesis is directly related to growth rate (40), and efficient ribosome assembly requires the coordinate synthesis of its components. We estimated growth rate differences along dendrites by measuring the spatiotemporal expression of an rRNA (*rrnB* operon) and a ribosomal protein (L33, encoded by *rpmGB*). For that purpose, we constructed an *rrnB-gfp* promoter fusion where *gfp* transcription was driven by the P1 and P2 *rrnB* promoters, and a translational *rpmGB-gfp* fusion containing the *rpmGB* promoter and Shine-Dalgarno sequences (23). The genes encoding both fusions were integrated in single copy at the *amyE* locus. Their expression was monitored with a stereo-fluorescence microscope at relatively low magnification (×15), which allowed semiquantitative measurements of an entire 1.5-cm dendrite with *quasi*–single-cell resolution. We observed very weak expression from the rRNA promoters (*rrnB-gfp*) at the base of dendrites, with a shallow gradient of increasing expression along the dendrite until a final steep increase in expression at the tip (Fig. 4a). A very similar pattern was obtained with *rpmGB-gfp* (L33), although with a rather constant low-level expression along the dendrite until a strong augmentation in expression within the last millimeter at the tip (Fig. 4b). The relatively high level of *rpmGB-gfp* fluorescence observed at the base is most likely due to the onset of bacterial multilayer formation that was perceptible at the periphery of the mother colony (Fig. 4b).

Importantly, these semiquantitative data were confirmed by quantitative *in situ* single-cell fluorescence measurements every 2.5 mm along the dendrite and at <0.5-mm intervals at the tip. The average fluorescence signal for the *rrnB-gfp* fusion in a single cell in the 1-mm tips was 1.2-fold higher than that in the population immediately behind (within 0.2 mm) and 2-fold higher than the signals obtained at the base of the dendrite (see Fig. S3a in the supplemental material). Moreover, the expression of the *rpmGB-gfp* fusion was reduced twofold in cells immediately behind the swarmers (see Fig. S3b).

10.1128/mBio.02102-16.4 FIG S3  Quantitative *in situ* analysis of *rrnB-gfp* and *rpmGB-gfp* expression in single cells. Fluorescence in single cells was measured based on images taken under identical conditions, i.e., ×1,000 magnification and constant exposure time at all positions within the dendrite (50 or 100 ms). Single-cell mean fluorescence intensities based on the analysis of at least 500 cells located at a given location are plotted against position along a 1.5-cm dendrite (plain line) and a 1-mm bud (dashed line); distance in both cases is measured from the edge of the mother colony. (a) Swarm of strain SSB2020, expressing the *rrnB-gfp* fusion construct. (b) Swarm of strain OMG981, expressing the *rpmGB-gfp* fusion construct. Error bars represent the standard deviations of the means. Download FIG S3, PDF file, 0.1 MB. Copyright © 2017 Hamouche et al. 2017 Hamouche et al. This content is distributed under the terms of the Creative Commons Attribution 4.0 International license.

We also measured *rrnB-gfp* and *rpmGB-gfp* expression in 1-mm predendrite buds that burst out from the edge of the mother colony 12 h after inoculation and represent the earliest observed stage of migration. Interestingly, the cells in the buds are swirling in vortices just like the cells in the dendrite tips. Quantitative analysis at four different positions across the 1-mm buds showed an almost constant high level of expression that was comparable to that in the swarmer cells at the tip of dendrites (see Fig. S3 in the supplemental material), a further indication that the buds are indeed composed of swarmers. A similar constant high level of expression of the *hag* gene across buds, comparable to that in dendrite tips, but much higher than that in stem cells, was found previously (18).

A more detailed analysis of the *rpmGB-gfp* fluorescence levels in individual cells showed heterogeneity of expression at the base of the dendrite that continued throughout the dendrite, with, however, an abrupt transition toward a unimodal expression pattern at the very tips of the community (see Fig. S4 in the supplemental material). This supports the view that the swarmers at the tip form a relatively homogenous population constituting the most active cells in the community with respect to replication and protein synthesis.

10.1128/mBio.02102-16.5 FIG S4  Swarmers are a unimodal population with respect to *rpmGB-gfp* expression. Monolayered 1.5-cm dendritic swarms of strain OMG981 were analyzed for the heterogeneity of *rpmGB-gfp* expression. High-resolution (×1,000) fluorescent images were taken *in situ* at various locations from the base to the tip as indicated. This figure shows the percentages of cells distributed over the fluorescence intensities measured within the population. A wide range of expression from the *rpmGB* promoter suggests the presence of metabolically more or less active subpopulations. The graph illustrates the transition to a unimodal population at the very tip of the bacterial community composed of the swarmer cells. Download FIG S4, PDF file, 0.2 MB. Copyright © 2017 Hamouche et al. 2017 Hamouche et al. This content is distributed under the terms of the Creative Commons Attribution 4.0 International license.

### Swarmer cells are actively making new cell walls and septa.

**FIG 5:**
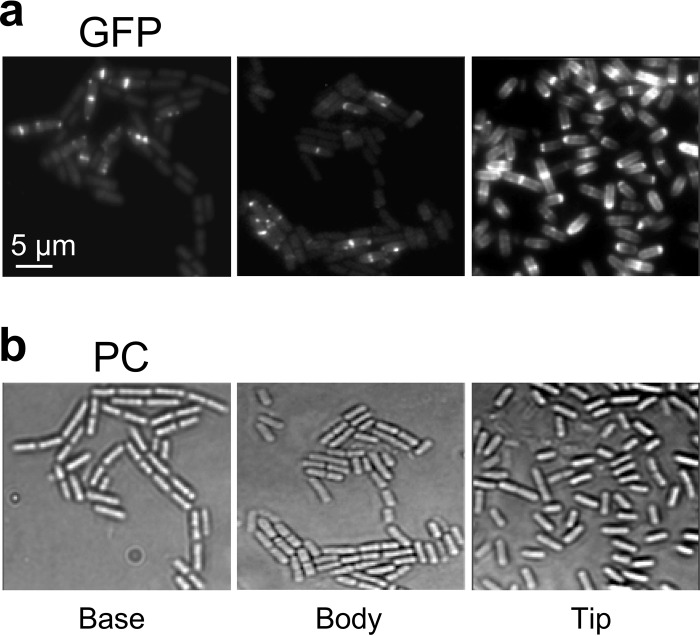
The swarmers leading the community are actively making cell wall. Cells selectively removed from the base, the body, and the tip of an advancing dendrite (strain SSB2026) were pulse labeled with 7-hydroxycoumarin-amino-d-alanine (HADA). Incorporation of HADA is indicative of active peptidoglycan synthesis. (a) Fluorescence images show that HADA preferentially labels cells at the tip, integrating into the division septum, daughter cell poles, and the elongating peripheral wall. (b) Phase-contrast control images.

Peptidoglycan (PG) is a major component of the cell wall, and bacterial growth and PG synthesis are tightly correlated (41, 42). d-Amino acids (DAA) present in short peptides cross-link the glycan strands that compose the polymer PG. Recently, fluorescent d-amino acids (FDAA) have been shown to be useful for probing *de novo* peptidoglycan synthesis and bacterial growth *in situ* (27, 43, 44). Regions of active growth can be tracked by the incorporation of FDAA in periplasmic transpeptidation reactions involved in PG biosynthesis (27, 43). Here, we used the fluorescent d-amino acid 7-hydroxycoumarin-amino-d-alanine (HADA) as a tool for the spatiotemporal determination of nascent PG sites within the migrating community. Cells removed from positions along dendrites were pulse labeled to incorporate HADA, revealing patterns that differed markedly with the position of the individual cells in the dendrite (Fig. 5a). More than 80% of the swarmer cells at the tip showed strong fluorescence at a single cell pole (presumably the new pole of a daughter cell following division) and scattered distribution of labeling at the peripheral wall (indicative of cell elongation) and/or at the division septum. These patterns are clearly associated with cell growth (45). In contrast, only about 30% of the cells located at the base and in the body of the dendrite incorporated HADA to a detectable level, the fluorescence intensity was lower than that seen in the swarmer cells, and fluorescence was almost never observed at the poles or in the peripheral wall (Fig. 5a). Moreover, the numbers of cells incorporating HADA at the base and in the body of the dendrite are likely overestimated due to a partial reactivation of growth during the 6-min labeling period in liquid medium. These results clearly support the notion that swarmers continue to grow and divide, while the stem cells show substantially reduced levels of cell wall production.

### Theoretical model of the advancing community.

**FIG 6:**
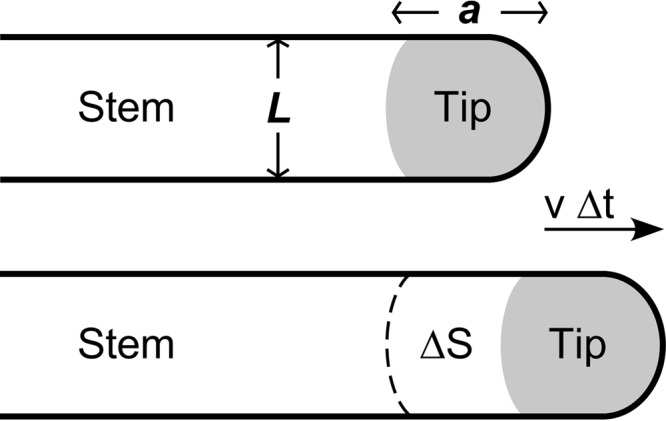
The linear expansion model. The sketch depicts the two-dimensional forward movement of a monolayered bacterial community. The dendrite is composed of two populations, the stem (immobile stem cells), with a width *L*, and a tip region extending over a length *a* that contains the swarmer cells. With a constant speed *v*, the swarmers in the tip cover new territory with a surface Δ*S* in a given time interval Δ*t*. The mathematical treatment of the advancing community, based on the sole multiplication of the swarmer cells, is given in “Theoretical model of the advancing community” in Results.

The combined experimental results clearly show that major cell cycle players are only active in cells in dendrite tips and, therefore, that only the swarmers are multiplying. This raises the possibility that the early monolayered dendrites at least are built primarily, if not only, from swarmer descendants. This implicitly indicates that half of the swarmer daughters are somehow left behind as relatively quiescent cells to form the dendrite stems, thus resolving the paradox that a constant rate of swarm expansion is not consistent with exponential growth of all cells in the community. To test these conclusions, we therefore attempted to construct a simple two-population mathematical model that might account for the linear growth of a dendrite when integrating the experimentally determined parameters (Fig. 6).

Consider a single dendrite extending only at its tip through the advancement of highly motile and dividing swarmer cells packed at a density of ρ_tip_. The remainder of the dendrite, from the mother colony to a distance *a* from the tip (Fig. 6), is populated by nondividing stem cells that exhibit no net motion along the dendrite.

During a short time interval, Δ*t*, the replication of the *N*_*S*_ swarmer cells produces Δ*N* = *N*_*S*_Δ*t*log_2_/*T* new cells, where *T* is the generation time. As *N*_*S*_ and the surface area (*aL*; in grey in Fig. 6) of the tip region remain constant during migration, statistically, half of all swarmer daughter cells are left behind (i.e., swarmer cells becoming stem cells) as the dendrite tip advances through a distance *v*Δ*t*, covering an area *LvΔt*. The density of cells in the newly formed segment of the dendrite is therefore expected to be ρ_stem_ = Δ*N*/*Lv*Δ*t* = ρ_tip_*a*log_2_/*vT*.

In the experiments, the tip length is of the order *a* = 1 to 2 mm, and the density ratio, ρ_tip_/ρ_stem_, is around 3 (Fig. 1) (18). According to our simple model, this ratio should be roughly equal to the ratio of the distance covered in one generation time over tip size, or *vT*/*a*log_2_. We assume that the generation time is the same as in liquid culture (*T* = 110 min). With an average speed of advancing dendrites of *v* = 1.8 mm/h (we measured 1.6 to 2 mm/h), the ratio *vT*/*a*log_*2*_ ranges from 4.8 to 2.4 when the tip size *a* is taken as 1 mm or 2 mm, respectively. The observed density ratio, ρ_tip_/ρ_stem_, ranges from 3.3 (*a* = 1 mm) to 2 (*a* = 2 mm). The calculated and experimentally determined values thus coincide best if we consider the metabolically active tip region to extend about 1.5 mm from the migration front. This is close to that of the swirling swarmer cell population, which is limited to about 1 mm by the decorrelation analysis (Fig. 1c). Nevertheless, it is remarkable that our quite-simple model that, for example, entirely neglects cell division behind the tip region, captures the experimentally measured movement of the bacterial community with surprising accuracy.

## DISCUSSION

We have shown that the tiny subpopulation of swarmer cells at the forefront of the migrating community replicates DNA, makes high levels of rRNA and a ribosomal protein, and synthesizes peptidoglycan, in marked contrast to the bulk of the cells in the dendrite stems. Swarmers occupy the nascent buds and then remain at the tip of the elongating dendrites, maintaining a constant surface area and population density. Therefore, we propose that looking backwards from the tip of a dendrite, one can see a gradient of swarmer descendants, the youngest near the tip and the oldest near the mother colony. Interestingly, this might be similar to the suggested sequence of events in a *Vibrio* swarm (46), based on cell morphology. When a swarmer cell divides, we propose that statistically, one daughter remains in the high-density population and the other daughter is left behind, quickly becoming immobilized. To explain the loss of mobility, we favor a quite simple physical hypothesis: the abrupt shift to a lower population density behind the tip means that the now relatively well separated individual cells are more exposed to the strong capillary forces prevailing in thin films, with consequent cessation of flagellar movement. Similarly, even swarmer cells at the leading edge of tips are so strongly clamped that they are transiently immobilized due to the asymmetry of the liquid meniscus (see Movie S1 in the supplemental material), until they are pulled inwards by the advancing community. Moreover, similar pinning of isolated cells at the swarm front, rendering them immobile until incorporated into a group, has been reported previously by several authors with different swarming species. Apparently, only collectively can the swarmers exert sufficient pressure on the tip border (47) to overcome the pinning forces and push out. This clamping process, as discussed below, might also explain the progressive growth arrest of the stem cells.

Interestingly, Sokolov and coworkers (48) showed that resuspension of *B. subtilis* 168 to high population densities was sufficient to promote the swirling vortices of cells that are characteristic of swarmers at the swarm front. Moreover, they found that the population density that is optimal for this collective swirling is probably the maximum compatible with maintenance of a fluid monolayer of cells. Indeed, in dendrite tips, when swarming is arrested (for example, when plates dry out), swarmer cells “crystallize” into confluent “mosaics” of tightly packed 5- to 6-cell rafts (20). Thus, the role of collective high-velocity swimming, dependent on the high population density, may simply be required to maintain the necessary fluidity of the swarm front in order to avoid stalling. However, how the swarmers preferentially push outwards is unclear, although it may be linked to the radial gradient in surfactin concentration (49), producing a wettability gradient, and to the fact that movement in the opposite direction is blocked by the immobilized stem cells. Additional experiments to further test our model could involve the use of mutant strains with a reduced growth rate under the same swarming conditions to check whether the model scaling still holds. It will also be useful to carry out experiments by varying the agar concentration and the degree of humidity (within a range of 20% to 80%). This will most likely affect some of the model parameters, like the tip length (i.e., swarmer population), which can be measured on time-lapse movies and, thus, checked as to whether they are still in agreement with the model.

In a number of bacteria swarming in thin fluid films as described in this study, it has been shown that flagella can act as mechanosensors to modulate gene expression and physiological functions (summarized in reference 3). However, there has been no report of growth inhibition in pinned cells, as seen here, in previous swarming studies. This may simply have been missed because the rapid multilayering of cells behind the swarming front under the fast-growing conditions used in other systems avoids surface tension effects on cells in upper layers. Moreover, in dendritic swarming in a monolayer, promoted perhaps by the low growth rate, surface tension clamping of the flagella or perturbation of the cell membrane might be sufficient to block or reduce the growth of the stem cells. On the other hand, we cannot exclude the possibility that *B. subtilis* swarmers divide asymmetrically, yielding one swarmer and one nonswarmer (i.e., a stem cell) that is reprogrammed to block key growth regulators, somewhat reminiscent of the division process observed in *Caulobacter* (50).

In contrast, we can exclude the possibility that the stem cells stop growing due to a lack of nutrients consumed by the preceding passage of the swarmers. If depletion were responsible for slow growth behind the tip, we should observe a strong influence when changing the quantity of available nutrients in the plate. This is not the case; the swarming phenotype remains unaltered even when the swarm plate medium is diluted up to 10-fold (data not shown). Moreover, sporulation (dependent on nutrient starvation) is never observed in dendritic swarming until 20 to 24 h of incubation, when the swarms are deeply multilayered (unpublished data). Finally, we note that when the dendrites reach approximately 1.5 cm in length, the switch to multilayered growth occurs dramatically, starting from the base, clearly indicating that nutrients are not limiting (23).

In summary, only the swarmers in the constant-volume, constant-population-density tips appear to be multiplying, while in some way, the consequent surplus of cells is left behind to form the bulk of the dendrite. Given the many characteristics that are conserved in different swarming systems, we suggest that this mechanism to produce biomass continuously from swarmer cell division, although different from the distinctive style of the *Proteus* model, may be found with other species and should now be tested. Finally, swarming is intimately linked to biofilm formation. A full understanding of the parameters guiding the swarming process is fundamental, because this process ultimately determines where the biofilm will be installed and, therefore, how we may eventually interfere with biofilm formation and dissemination.

## MATERIALS AND METHODS

### Bacterial strains and growth conditions.

Strain SSB2026 was the parent strain (*trpC2 thrC*::*sfp*^+^* erm*) of most strains used in this study. This is an *swrA*^+^ revertant of strain OMG930 (23). Strain SSB2041 (*trpC2 swrA*^+^* thrC*::*sfp*^+^* erm amyE*::*P*_*xyl*_*gfp-DnaA cat*) expressing an inducible GFP-DnaA fusion protein was obtained by transforming SSB2026 with plasmid pAS16 (31), integrated at the *amyE* locus. Strain SSB2022 (*trpC2 thrC*::*sfp*^+^* erm PdnaN-gfp-dnaN cat*) expressing an ectopic GFP-DnaN fusion protein was constructed by transforming SSB2026 cells with chromosomal DNA from strain JWV201 carrying the *PdnaN-gfp-dnaN* fusion construct (36, 37). Strain OMG981 (*trpC2 swrA thrC*::*sfp*^+^* erm amyE*::*PrpmGB-gfp mut3 spc*) was described earlier (23); at 70% humidity, its swarming kinetics and patterns are very similar to those of the undomesticated strain 3610. SSB2020 (*trpC2 swrA thrC*::*sfp*^+^* er*, *amyE*::*PrrnB-gfp spc*) was obtained by transforming OMG930 with the *amyE* integrative plasmid pHMrrnB1.

Strains were grown in liquid synthetic B medium (51) with aeration at 37°C. For threonine-auxotrophic strains, threonine was added to a final concentration of 1 mM. Antibiotics were added to agar plates at the following final concentrations: chloramphenicol, 5 μg/ml; spectinomycin, 100 μg/ml; and erythromycin-lyncomycin, 0.5 μg/ml and 12.5 μg/ml.

### Plasmid construction.

pHMrrnB1 was constructed by amplifying the *rrnB* promoter region, starting 122 bp upstream from the P1 promoter, using oligonucleotides HP1538 (5′ AGGTCGAATTCAAACAACAAGATCACATGACTGATG) and HP1539 (5′ GTCACGATATCTTTCATTTTAGAACACTCCTTATTCATTGTTGTAGCTTTACTAATATAACATTCAGCA). The resulting 298-bp fragment was cleaved with EcoRI and EcoRV and inserted into the respective sites of the GFP fusion vector pDL30-gfpmut3-ter (23).

### Conditions for dendritic swarming experiments.

For swarming on B medium, cultures for inoculation were prepared in 3 ml LB medium inoculated from a single colony on an LB agar plate and shaken for at least 3 h at 37°C. The culture was diluted to an optical density at 570 nm (OD_570_) of ~0.0075 in B medium and shaken overnight at 30°C. In the morning, the still-exponentially growing overnight culture was diluted to an OD_570_ of ~0.1 and grown at 37°C into stationary phase. The culture was diluted, and 10^4^ CFU (2 μl) was placed at the center of a 9-cm swarm plate containing 25 ml of B medium (0.7% Bacto agar) prepared 1 h before inoculation, and the plates dried with lids open for 5 min in a laminar flow hood. The plates were incubated at 30°C in a Climacell 111 incubator with a relative humidity of 40% for *swrA*^+^ strains and 70% for the *swrA* frameshift mutant strain OMG981 (18) until the dendrites reached approximately 1.5 cm in length (~18 to 19 h). In order to obtain robust, sustained swarming on B medium, it was important to pay careful attention to the level of humidity. The precise control of temperature and humidity guaranteed reproducible timing of the initiation of migration and pattern formation and avoided any residual free water on the agar surface. Thus, a drop of water placed on these swarm plates did not spread (52). Twelve hours after growth of the central MC, 8 to 12 monolayered primary dendrites radiate outwards from initial buds forming at the edge of the MC, at a constant speed of approximately 2 mm/h (0.6 μm/s) at 30°C. 

### Imaging and single-cell analysis.

Fluorescence imaging and visualization of cells were done either with a stereomicroscope (Zeiss Lumar) at ×15 magnification or using various objectives fitted on a phase-contrast/fluorescence microscope (Zeiss Axio Imager M1). Both microscopes were equipped with an AxioCam MRm camera (Zeiss). GFP fluorescent images with the Lumar instrument were taken using filter set 38 HE eGFP (Zeiss), and filter set 10 (Zeiss) was used with the Axio Imager M1. High-magnification *in situ* images were obtained with a 63× air or a 100× oil objective. For the latter, we gently placed a coverslip over regions of dendrites to be observed. This ensured minimal disturbance of the sample with the displacement of, at most, a few cells from the edge of a dendrite. Image acquisition was done with AxioVision software (release 4.6.3). Semiquantitative analysis of fluorescence intensity along an entire dendrite was carried out on false-colored images in order to visualize the relative levels. Calculation and formatting of the fluorescence intensities were carried out using ImageJ software (53).

Quantitative analysis of fluorescence signals (dispersed or in foci) was done on high-resolution false-colored images using the AutoMeasure module associated with the AxioVision software. When measuring the intensity of foci, local background fluorescence was measured within the individual bacteria and subtracted. The fluorescence distribution within a given population was analyzed as described previously (18).

For the visualization of cells from exponentially growing and stationary-phase cultures, overnight cultures in B medium were diluted to an OD_570_ of ~0.1 and grown at 37°C in fresh B medium for at least three generations (exponential phase) and to *T*4 (4 h after the transition from exponential growth) for stationary phase. Cells were mounted on 1% (wt/vol) agarose pads, and images acquired by an AxioCam MRm camera (Zeiss) using a 1.3 numeric aperture (NA) 100× oil objective on a phase-contrast/fluorescence microscope (Zeiss Axio Imager M1).

For the examination of fluorescence intensity of DnaA foci, cells were scraped from different regions of the dendrites (edge of mother colony, base, dendrite body, and tip) and dispersed in B medium. One microliter of the cell suspension was spotted on 1% (wt/vol) agarose pads and gently covered by a new coverslip. Cells were imaged using a phase-contrast/fluorescence microscope (Zeiss AxioImager M1) with a 1.3 NA 100× oil objective. Quantitative single-cell analysis of the fluorescence intensity of DnaA foci was performed using ImageJ software. The fluorescence intensity of GFP-DnaA foci was analyzed along at least 40 dendrites from three independent experiments, totaling 1,008 cells from the base, 1,246 cells from the body, and 1,213 cells from the tip of the dendrites to be analyzed.

### Measurement of population density.

Population densities were measured *in situ* in entirely monolayered 1- to 1.5-cm dendrites. Images were captured using a 40× Neofluar objective mounted on an AxioImager M1 microscope (Zeiss). Images were processed using ImageJ software. The calculated population densities were confirmed manually by counting at least 2,000 cells in three adjacent fields. Cells containing a clear septum were counted as two cells. In order to obtain sharp images of hypermobile cells in the tip region, swarms were placed under the microscope at room temperature without a coverslip for 5 min to reduce mobility.

### Fluorescent d-amino acid staining of the peptidoglycan.

Staining with 7-hydroxy-coumarin-amino-d-alanine (HADA) was used to track regions of active peptidoglycan synthesis (43). Swarms of strain SSB2026 were established up to about 1.5 cm, in order to maintain a cellular monolayer over the entire dendrite, minimizing any multilayering during the time required for measurements. Cells were removed from several dendrites at different positions (base, body, and tip) and resuspended in B medium containing 1 mM HADA and 1% (vol/vol) dimethyl sulfoxide (DMSO). Cells were incubated for 6 min at 30°C with rocking, washed three times with 1× phosphate-buffered saline (PBS) buffer, and spotted on 1% (wt/vol) agarose pads (0.2 g agarose in 20 ml 1× PBS). The cells were gently covered by a coverslip, and the edges sealed with a 1:1:1 mixture of melted petroleum jelly, lanolin, and paraffin before imaging (as described in reference 27). Approximately 4,000 cells in each region were analyzed.

### Decorrelation analysis.

For the decorrelation analysis, an elongating 5-mm dendrite was filmed for 1.1 s (50 images) in four different zones—at the tip and at 1, 4, and 4.5 mm from the tip. For each image of the series, the pixel values were multiplied by the corresponding pixel values of the first image. The average pixel values obtained after this operation will be greater the more an image resembles the first image. Plotting these mean values in a chronological order results in exponentially decreasing (decorrelation) curves. The characteristic decorrelation time obtained is a quantitative measure of the mobility of the bacteria in a short time lapse.
