# Supplementary material for: Bacillus subtilis Swarmer Cells Lead the Swarm, Multiply, and Generate a Trail of Quiescent Descendants
Source: mBio. 2017 Feb 7;8(1):e02102-16. doi: 10.1128/mBio.02102-16 (PMC5296600; doi:10.1128/mBio.02102-16)
Supplement: FIG S1 [file mbo001173183sf1.pdf]

Supplementary Figure 1

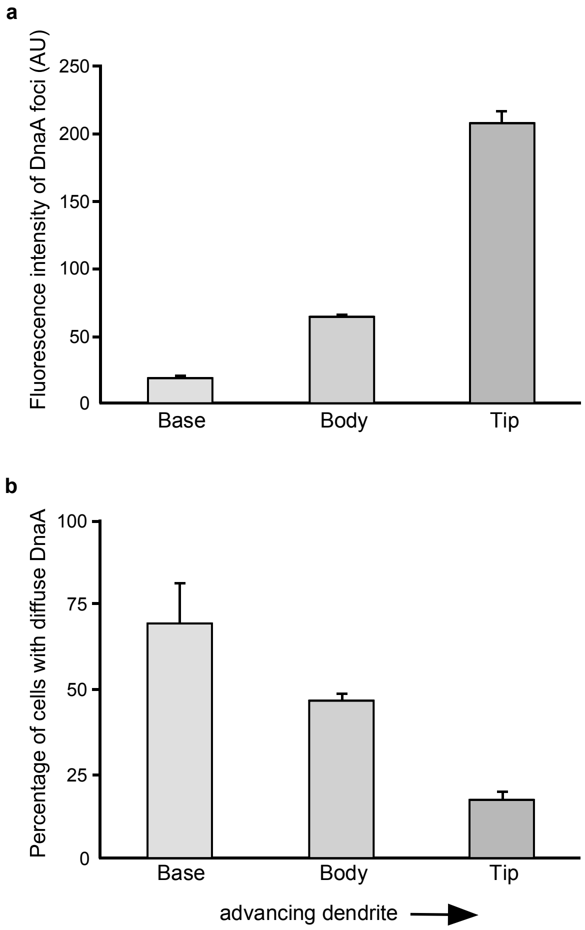

Supp. Fig. 1. Expression and distribution of the replication initiator protein DnaA. (a) Mean fluorescence intensity of DnaA foci observed in individual cells localised at the base, body and tip of the swarming community. (b) Percentage of cells that show a diffuse DnaA expression pattern and no visible GFP-DnaA foci. Error bars represent the standard deviation of the mean. AU = arbitrary units.
