# Supplementary material for: Bacillus subtilis Swarmer Cells Lead the Swarm, Multiply, and Generate a Trail of Quiescent Descendants
Source: mBio. 2017 Feb 7;8(1):e02102-16. doi: 10.1128/mBio.02102-16 (PMC5296600; doi:10.1128/mBio.02102-16)
Supplement: FIG S2 [file mbo001173183sf2.pdf]

**Supplementary Figure 2**

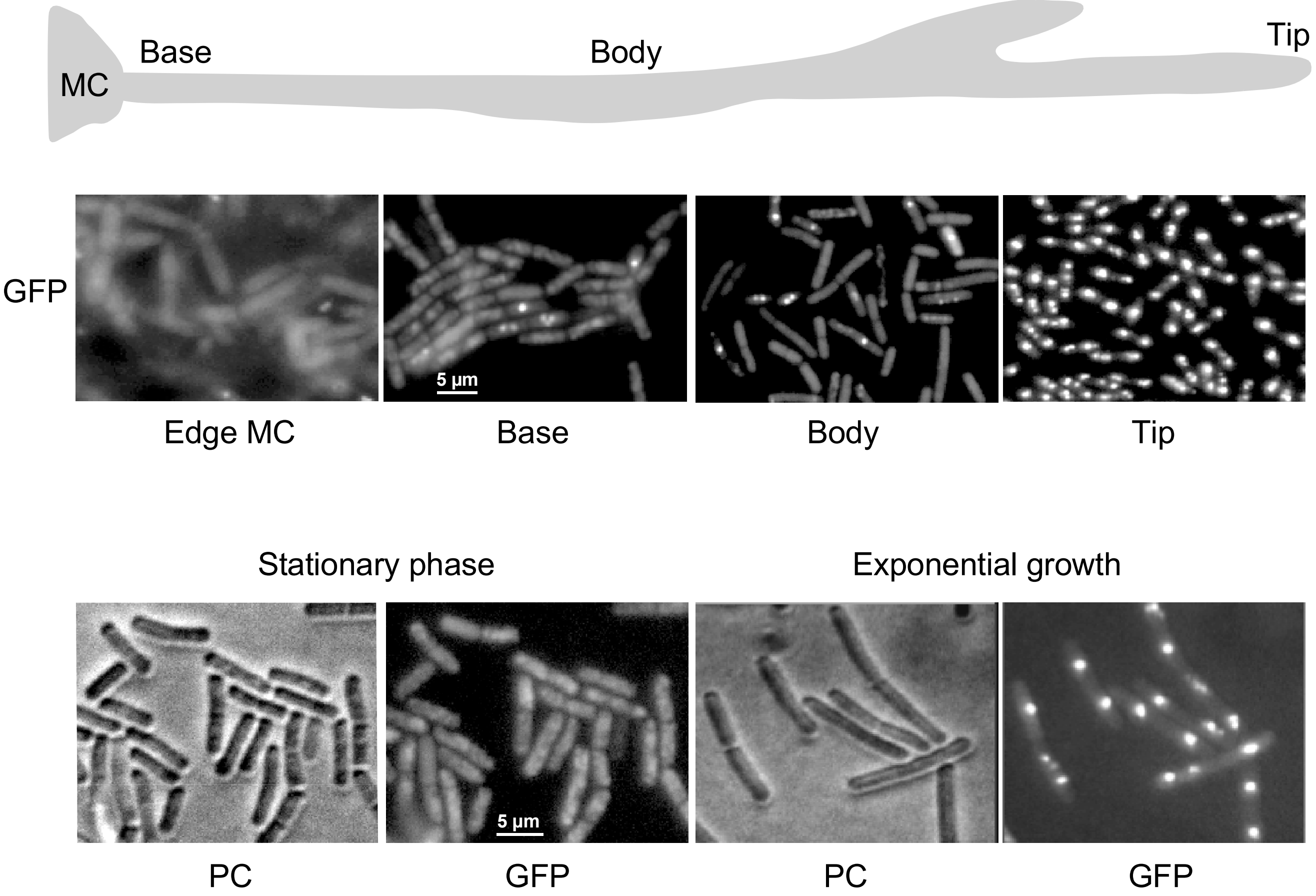


**Supp. Fig. 2. GFP-DnaN expression in the swarm and liquid culture.** Upper panel : *In situ* fluorescence microscopy images (100x) of a 1.5 cm swarm expressing a fluorescent GFP-DnaN fusion protein (strain SSB2022). The images taken in the body and at the tip of the dendrite are identical to those shown in Fig. 3.

The lower panel shows a control experiment where strain SSB2022 was grown in liquid culture (B-medium). Cells were taken either during exponential growth or from stationary phase and GFP-DnaN expression analysed by fluorescence microscopy under the same conditions as the swarming cells. The PC labeled images show the same cells imaged by phase contrast microscopy.
