# Supplementary material for: Bacillus subtilis Swarmer Cells Lead the Swarm, Multiply, and Generate a Trail of Quiescent Descendants
Source: mBio. 2017 Feb 7;8(1):e02102-16. doi: 10.1128/mBio.02102-16 (PMC5296600; doi:10.1128/mBio.02102-16)
Supplement: FIG S3 [file mbo001173183sf3.pdf]

Supplementary Figure 3

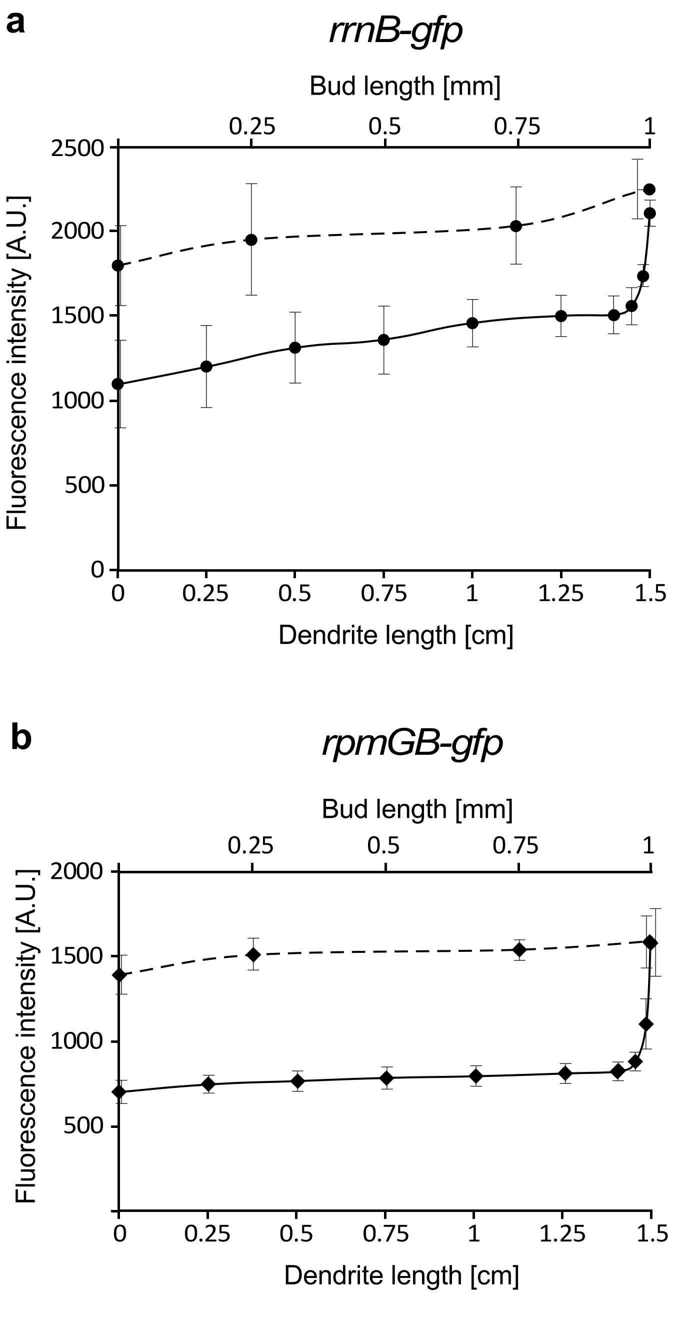

SuppFig. 3. Quantitative *in situ* analysis of *rrnB-gfp* and *rpmGB-gfp* expression in single cells. Fluorescence in single cells was measured based on images taken under identical conditions, i.e. x1000 magnification and constant exposure time at all positions within the dendrite (50 or 100 msec). Single cell mean fluorescence intensity based on the analysis of at least 500 cells located at a given location is plotted against the position along a 1.5 cm dendrite (plain line) and 1 mm bud (dashed line); distance measured in both cases from the edge of the mother colony. (a) swarm of strain SSB2020, expressing the *rrnB-gfp* fusion construct. (b) swarm of strain OMG981, expressing the *rpmGB-gfp* fusion construct. Error bars represent the standard deviation of the mean.
