# Supplementary material for: Bacillus subtilis Swarmer Cells Lead the Swarm, Multiply, and Generate a Trail of Quiescent Descendants
Source: mBio. 2017 Feb 7;8(1):e02102-16. doi: 10.1128/mBio.02102-16 (PMC5296600; doi:10.1128/mBio.02102-16)
Supplement: FIG S4 [file mbo001173183sf4.pdf]

Supplementary Figure 4

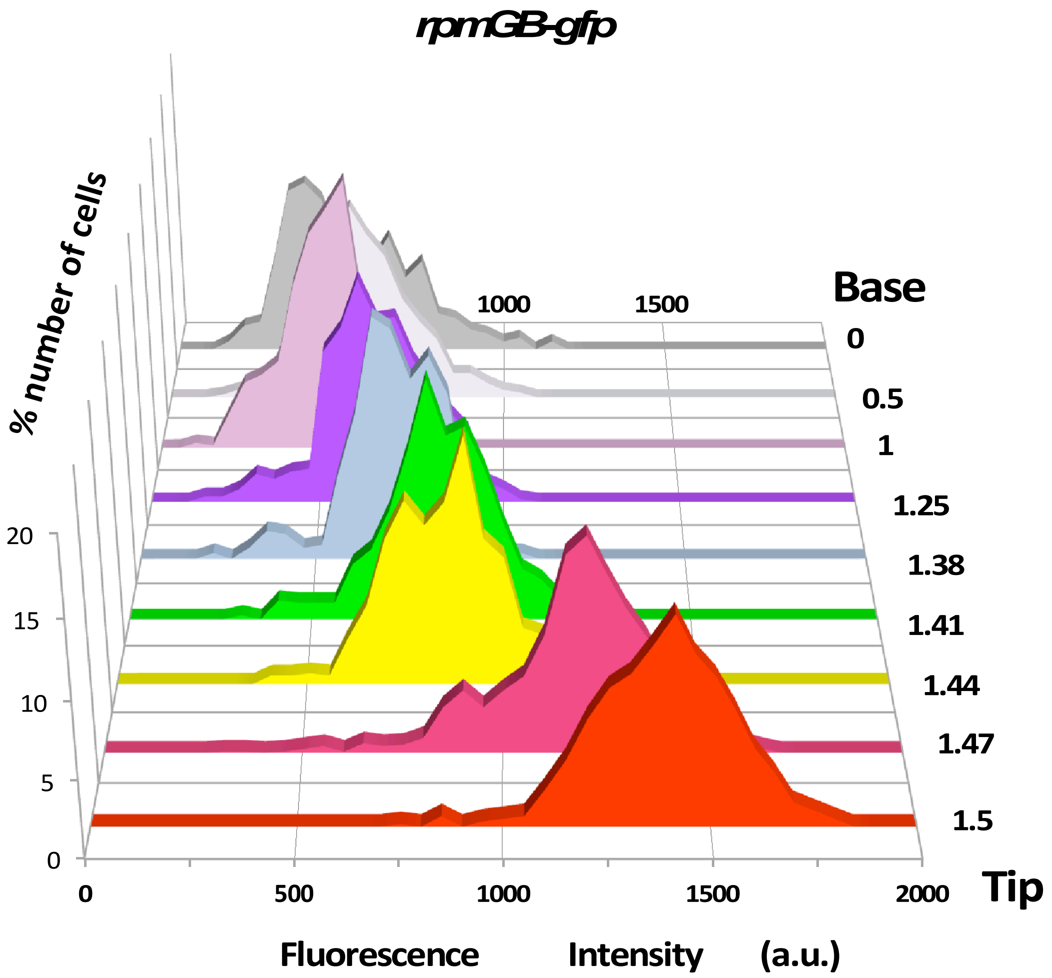

SuppFig. 4. Swarmers are a unimodal population with respect to *rpmGB-gfp* expression. Monolayered 1.5 cm dendritic swarms of strain OMG981 were analysed for the heterogeneity of *rpmGB-gfp* expression. High resolution (1000x) fluorescent images were taken *in situ* at various locations from the base to the tip as indicated. This figure shows the percentage of cells distributed over the fluorescence intensity measured within the population. A wide range of expression from the *rpmGB* promoter suggests the presence of metabolically more or less active sub-populations. The graph illustrates the transition to a unimodal population at the very tip of the bacterial community composed of the swarmer cells.
